# Caseous Lymphadenitis Outbreak in Dairy Cattle: Clinical Findings, Management, and Autogenous Vaccine Development

**DOI:** 10.3390/vetsci12121155

**Published:** 2025-12-03

**Authors:** Lina Costa, Hélio Correia, João Costa

**Affiliations:** 1 CLILEGRE—Veterinary Clinic of Portalegre, 7300-817 Portalegre, Portugal; correia.hbf@gmsil.com (H.C.); hvclilegre@gmail.com (J.C.); 2 Elvas School of Biosciences, Polytechnic University of Portalegre, Edifício Quartel do Trem, Avenida 14 de Janeiro nº21, 7350-092 Elvas, Portugal; 3 VALORIZA—Research Centre for Endogenous Resource Valorization, Polytechnic University of Portalegre, Edifício BioBIP, Campus Politécnico, 10, 7300-555 Portalegre, Portugal

**Keywords:** autovaccine, *Corynebacterium pseudotuberculosis*, dairy cattle, outbreak, Portugal

## Abstract

Caseous lymphadenitis (CL), caused by *Corynebacterium pseudotuberculosis*, typically affects small ruminants but is rarely reported in cattle. This case report describes an outbreak of CL in a dairy herd in southern Portugal, where 55 of 500 animals (11%) were clinically affected. Diagnosis was based on characteristic lesions and laboratory confirmation by bacterial culture and PCR. Control measures included isolation, culling, environmental disinfection, and vector management. An autogenous vaccine was prepared from herd isolates and administered under veterinary supervision. No further clinical cases occurred following vaccination. Although immunological and efficacy assessments were not performed, the apparent control of the outbreak suggests that autogenous vaccination, combined with strict biosecurity, may support disease management in atypical hosts. This report underscores the importance of recognizing *C. pseudotuberculosis* infections in cattle and highlights the need for further evaluation of autogenous vaccines under controlled conditions.

## Simple Summary

This case report describes an outbreak of caseous lymphadenitis (CL) in a dairy herd of 500 Friesian cows in Évora, southern Portugal—an unusual event, as CL typically affects small ruminants. Fifty-five cows (11%) developed swelling of lymph nodes, weight loss, fever, and reduced milk production, with lesions most frequently observed around the head. *Corynebacterium pseudotuberculosis* was identified as the causative agent by bacterial culture and PCR. The outbreak was likely facilitated by poor biosecurity, close animal contact, shared equipment, and proximity to infected small ruminants. Control measures included isolation and culling of affected animals, environmental disinfection, and vaccination of the herd using an autogenous vaccine prepared from the outbreak strain. Although vaccination is recognized as a potential control strategy against caseous lymphadenitis, no commercial vaccines are currently available in Europe for ruminants, limiting the feasibility of this approach under current regulatory conditions. This case highlights the underestimated risk of CL transmission across species and underscores the importance of surveillance, biosecurity, and vaccination strategies to prevent the spread of this disease in regions where it is endemic among small ruminants.

## 1. Introduction

Caseous lymphadenitis (CL) is an infectious disease typically associated with small ruminants such as sheep and goats, caused by *Corynebacterium pseudotuberculosis*. In recent years, however, there have been reports indicating that other animal species, including cattle, horses, and pigs, are also susceptible to this pathogen [1,2,3,4,5,6]. This has led to a significant increase in the disease’s economic impact. In Portugal, CL is endemic in small ruminants. An author study of 2021 [7] reports a farm-level seroprevalence of 75.4% in Portugal, where the disease is not subject to specific control or prevention measures [8]. Yeruham et al. (2004) reported *C. pseudotuberculosis* infection in Israeli dairy cattle, where the condition was manifested in cutaneous, mastitis and visceral forms [1]. Steerforth & Marutsov (2017) described the pathogenesis of a herd case of ulcerative lymphangitis in Bulgarian Holstein–Friesian cows with specific cutaneous lesions on the hindlegs, multitudinous abscessations, ulcerative lymphangitis and mastitis [6]. However, there is a paucity of literature on caseous lymphadenitis in cattle worldwide. Moreover, to our knowledge, there is no report of the disease in cattle in Portugal. Although the disease is endemic in some regions, outbreaks in dairy cattle herds are relatively rare [9] but very prevalent in sheep and goats [7].

*C. pseudotuberculosis* is the causative agent of CL in cases reported in ruminants and horses, but the strains involved belong to different genetic and biochemical serotypes. Two biotypes of *Corynebacterium pseudotuberculosis* are recognized based on their nitrate-reducing capacity: nitrate-negative strains, classified as serotype I (*ovis biotype*), and nitrate-positive strains, designated as serotype II (*equi biotype*). Strains isolated from sheep and goats are typically nitrate-negative, whereas those from horses are predominantly nitrate-positive. Isolates derived from cattle exhibit variable nitrate-reducing capabilities [10,11]. Oedematous Skin Disease (OSD), which is endemic among buffalo in Egypt, is primarily attributed to *C. pseudotuberculosis* serotype II (*equi biotype*; nitrate-positive) [12]. More recently, molecular and phylogenetic tools such as multilocus sequence typing (MLST) [13] and Enterobacterial Repetitive Intergenic Consensus PCR (ERIC-PCR) [14] have been employed to differentiate strains of *C. pseudotuberculosis*. Evidence suggests substantial genetic divergence between the *ovis* and *equi* biotypes [10,13], as well as notable genetic diversity among strains isolated from different host species and geographic regions [14]. A pangenomic analysis involving 15 *C. pseudotuberculosis* strains has been completed [10], and Almeida et al. (2016) reported the genome sequence of a highly virulent strain, VD57 (*Cp_VD57*) [15]. Although the nucleotide-level modifications contributing to differential virulence remain unidentified, current research efforts are focused on elucidating the genes differentially expressed in in vitro cultures and within granulomatous lesions [15]. The mechanisms of transmission, pathogenic processes, and economic implications of *Corynebacterium pseudotuberculosis* infection in cattle remain inadequately characterized. Although the bacterium exhibits a broad host range, natural interspecies transmission between small ruminants and cattle appears to be infrequent. Nevertheless, both biotype I (*ovis*) and biotype II (*equi*) have been isolated from bovine cases [10]. The clinical and epidemiological characteristics of the outbreak described in this study are suggestive of infection with the *equi* biotype (biotype II) of *Corynebacterium pseudotuberculosis*; however, biotype determination through genetic or biochemical characterization was not performed.

As this case report demonstrates, the lack of effective control strategies in these populations increases the risk of the disease spreading to other animals. This report describes an unusual outbreak of CL in a dairy cattle herd in the Portuguese Évora region, highlighting the disease’s potential to impact livestock beyond its traditional hosts. The main purpose of this work is to examine a sudden outbreak in a dairy farm in Portugal and identify the possible contributing factors and how to control and prevent the spread of the disease.

## 2. Case Description

The disease occurred on a family-owned dairy farm in the Évora region, southern Portugal, which has been operational for more than 10 years. The farm houses approximately 500 Frisian cows, with the primary focus on milk production. The herd was previously healthy and had not experienced any prior incidences of CL or related diseases.

**Figure 1:**
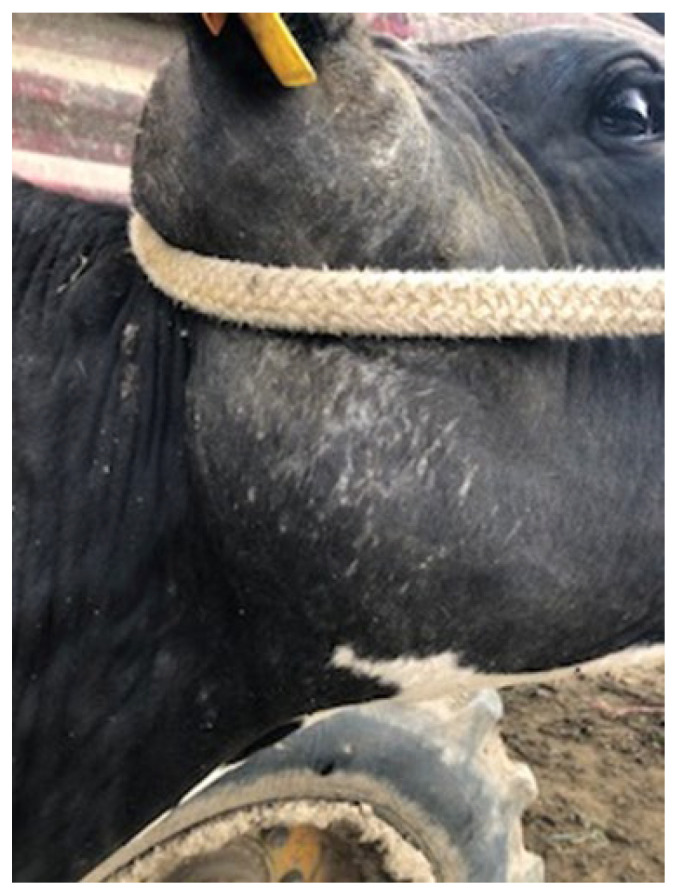
Enlarged submandibular lymph node.

**Figure 2:**
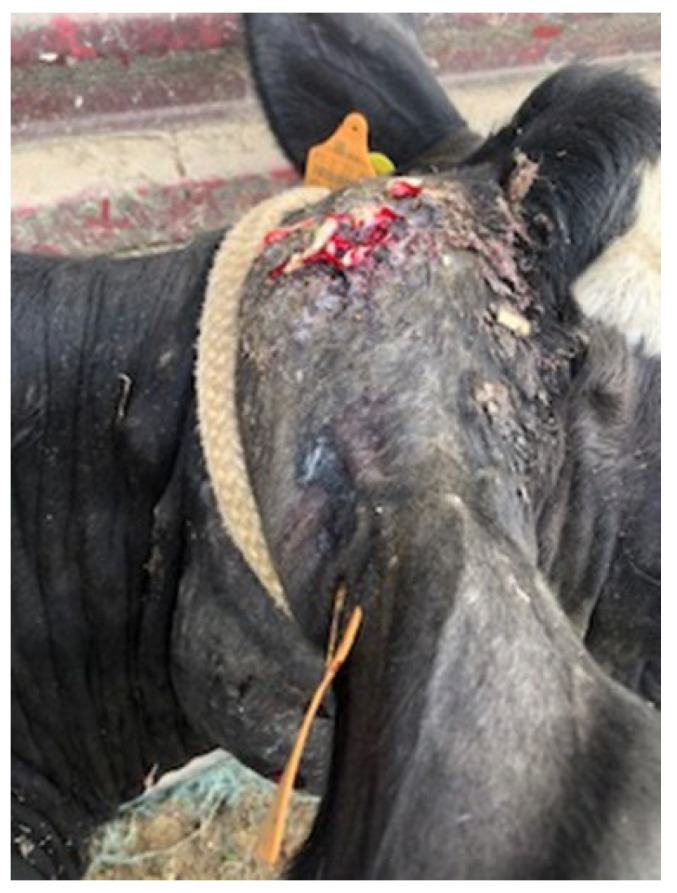
A close-up image of pyogranulomatous lesions in the head region and caseous material.

**Figure 3:**
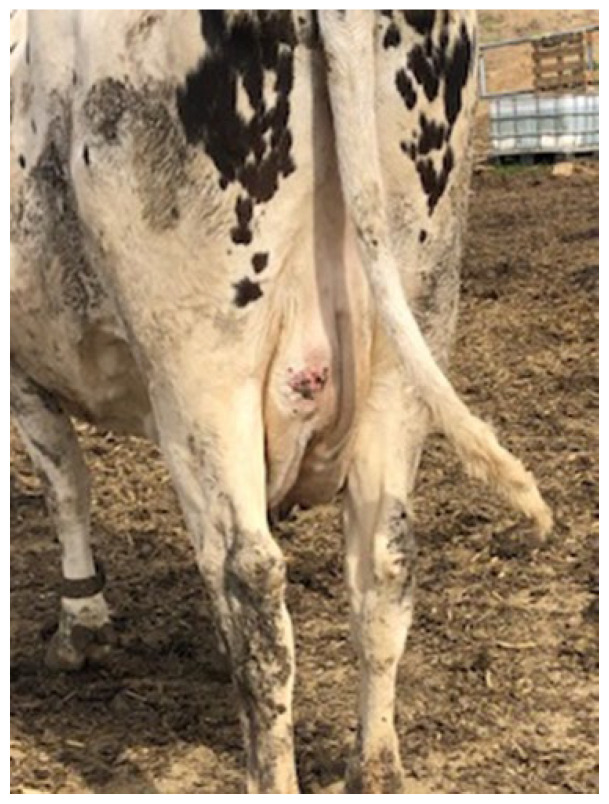
Enlarged retromammary lymph node.

The outbreak began in late February 2022 when several cattle were observed with swollen lymph nodes. The disease affected 55 cattle, primarily adult females, with a few cases also reported in young heifers. The clinical signs included visible abscesses, particularly in the submandibular and cervical lymph nodes (Figure 1), head regions (Figure 2) and retromammary (Figure 3), and reduced milk yield, while some animals exhibited weight loss, along with intermittent fever and loss of appetite. During the follow-up period, affected animals exhibited progressive weight loss and a noticeable reduction in milk production (milk production dropped by approximately 18% during the peak of the outbreak). These changes were reported by the producer and reflect the clinical deterioration observed at the herd level. As this was a field case description rather than a controlled research study, systematic quantitative records (e.g., mean body weight or milk yield with standard deviation) were not available; the information should therefore be interpreted as qualitative field evidence. Approximately 11% of the herd was clinically affected.

Prior to the outbreak, no routine screening for *C. pseudotuberculosis* had been implemented on the farm. This underscores the importance of regular surveillance even in closed herds.

## 3. Diagnosis

Pyogranulomatous lesions are commonly associated with chronic infectious conditions such as actinobacillosis, tuberculosis, and cutaneous abscesses caused by *Staphylococcus aureus* and *Actinomyces pyogenes*. In the case of caseous lymphadenitis (CL), although *Corynebacterium pseudotuberculosis* is recognized as the principal etiological agent, the presence of other pyogenic bacteria should not be overlooked. Pathogens such as *S. aureus*, *Trueperella pyogenes*, and *Pasteurella multocida* have been isolated from CL lesions, suggesting possible co-infections or secondary bacterial colonization that may complicate disease progression and therapeutic management [16,17,18].

**Figure 4:**
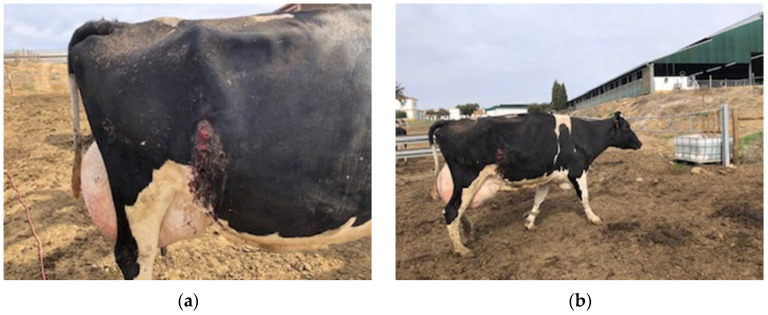
Enlarged and abscessed lymph nodes (**a**), weight loss (**b**).

**Figure 5:**
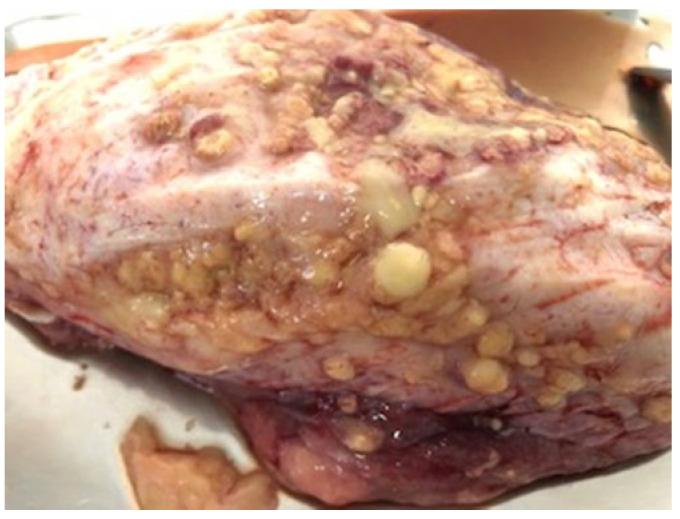
Post-mortem examination revealed the presence of characteristic caseous exudate within lymph nodes and internal organs. The image illustrates pyogranulomatous and caseous necrotic lesions localized in the thoracic pleural cavity, consistent with systemic caseous lymphadenitis.

Based on clinical signs—including enlarged and chronic pyogranulomatous lymph nodes, progressive weight loss (Figure 4a,b), and decreased milk production—a preliminary diagnosis of caseous lymphadenitis (CL) was established. Post-mortem examination of two affected animals revealed characteristic caseous necrotic lesions in both lymph nodes and internal organs (Figure 5), consistent with CL. Samples (pyogenic and pyogranulomatous) collected from 5 animals were sent to a commercially accredited veterinary diagnostic laboratory. Bacterial cultures obtained from pyogranulomatous lesions yielded *Corynebacterium pseudotuberculosis*, confirming the etiological agent. No other bacterial pathogens were identified. Additionally, conventional PCR targeting the *pld* gene (phospholipase D) of *Corynebacterium pseudotuberculosis,* following the protocol established by Pacheco et al., 2007 [19], was conducted to confirm the culture results. The successful isolation of the bacterium enabled the development of an autogenous vaccine for herd-specific immunization.

Advanced diagnostic approaches, such as molecular biotyping or antimicrobial susceptibility testing, were not available in the laboratory at the time of the outbreak and therefore could not be performed. As this investigation was carried out in the context of a field case rather than a controlled research project, the diagnostic process necessarily relied on routine methods.

## 4. Management and Treatment

Because of the persistent nature of the infection and the widespread prevalence within the herd, managing this *C. pseudotuberculosis* outbreak proved to be a significant challenge. Practical observations from caseous lymphadenitis outbreaks in small ruminants indicate that in herds with a high rate of infection, a combination of vaccination and strict hygiene protocols is likely to be effective.

Upon diagnostic confirmation, the affected animals were immediately isolated to prevent further transmission, and culling was carried out within one week of diagnosis. In line with recommendations for CL outbreaks in larger livestock populations, the decision was made to cull the infected animals to prevent further spread of the disease to the healthy members of the herd.

*Corynebacterium pseudotuberculosis* is recognized as a highly resilient microorganism. In sheep, individuals presenting with the cutaneous form of caseous lymphadenitis and superficial necrotic lesions serve as significant sources of environmental contamination. These lesions can release large quantities of viable bacteria—ranging from 10^6^ to 5 × 10^7^ cells per gram—onto the skin and wool, facilitating transmission to other animals [20]. The pathogen’s environmental persistence further exacerbates the risk of spreading, with documented survival for up to six months in the environment [21] and up to 55 days in organic matter [18]. Comprehensive biosecurity measures were implemented, including the disinfection of facilities and equipment that could have been contaminated by the infected animals, as well as better cleaning and disinfection protocols, to reduce the risk of further infection. The use of fly control measures was implemented, as flies can transfer the bacteria from infected animals to healthy ones, especially when there are open abscesses or wounds on the skin of affected animals.

As antibiotic therapy at the herd level was not feasible—due to excessive costs (including milk withdrawal), limited effectiveness, and the requirement for extended treatment durations—the cornerstone of our management strategy was autogenous vaccination.

The autogenous vaccine was produced and administered in compliance with national regulations governing veterinary autovaccines. It was formulated in a licensed veterinary laboratory (EXOPOL SL, San Mateo de Gállego, Zaragoza, Spain) using *Corynebacterium pseudotuberculosis* isolates obtained from clinically affected animals. Based on information provided by the manufacturer, a 24 h culture of *C. pseudotuberculosis* (sourced from the same farm) containing 9.0 × 10^8^ colony-forming units/mL was inactivated using 0.4 mL/100 mL of formalin. Aluminum hydroxide (Alhydrogel, Brenntag Biosector^®^) was then added as an adjuvant to formulate the inoculum.

The inactivated autogenous bacterin vaccine was tested and administered to a limited number of clinically healthy animals following the initial outbreak in 2022. Following standard guidelines provided by the vaccine manufacturer, per protocol, a 4 mL dose of this vaccine was delivered subcutaneously into the thoracic skin fold, just behind the elbow, with a booster dose administered 21 days later. No adverse reactions were observed. Subsequently, all animals on the premises received the vaccine. The youngest animals vaccinated were 12-month-old heifers. A follow-up vaccination was conducted four months later, with a second booster administered six months after that.

The autogenous vaccination protocol was repeated in 2023 as a preventive measure against further cases of caseous lymphadenitis. All incoming replacement animals are enrolled in the vaccination program upon reaching 12 months of age. Although no serological testing was performed, the herd was monitored clinically for 12 months following vaccination. During this period, no new clinical cases were detected, suggesting field-level effectiveness of the autogenous vaccine. As of the latest monitoring period in 2024, no further clinical cases of caseous lymphadenitis have been reported.

## 5. Discussion

This report details an outbreak of caseous lymphadenitis (CL) in cattle on a farm in the Évora region of Portugal, highlighting the disease’s capacity to affect species beyond its usual hosts—sheep and goats. While CL is endemic in small ruminants in Portugal, its emergence in cattle underscores the multispecies potential of *Corynebacterium pseudotuberculosis* and the need for increased vigilance across livestock operations.

Several factors likely contributed to the outbreak. The farm’s humid climate and high animal density created favorable conditions for disease transmission. *C. pseudotuberculosis* spreads through direct contact with infected material, via skin wounds, contaminated equipment, or by flies [7]. Close contact between animals, especially around shared feeding and watering areas, may have further facilitated the spread. Additionally, the presence of both sheep and goats, along with shared grazing areas, likely accelerated cross-species transmission.

Most clinical cases and suspected lesions were observed in dairy cows that regularly congregate in the milking parlor, a setting that facilitates close physical contact and increases the likelihood of exposure to open pyogranulomatous lesions—presumably the primary route of transmission. Nevertheless, aerosol transmission cannot be excluded, as pleural lesions were identified during necropsy. In sheep, the respiratory route is well-documented in the literature as a plausible and significant mode of transmission [20], suggesting a similar possibility in cattle.

The farm’s lack of prior isolation protocols for incoming animals and equipment also presented opportunities for the pathogen’s introduction. Inadequate biosecurity measures and the absence of vaccination programs for cattle are further possible contributors. These issues reflect a broader challenge in Portugal, where CL control efforts, although scarce, have traditionally focused on small ruminants, leaving cattle vulnerable to outbreaks.

This case highlights the underappreciated risk of CL affecting cattle and other livestock species. It calls attention to the importance of implementing comprehensive biosecurity protocols in Portuguese livestock farms, including isolation of new or symptomatic animals, improved hygiene, and the control of potential vectors such as flies. This atypical host occurrence constitutes the primary novelty of the study, as it highlights the need for veterinarians to consider CL in the differential diagnosis of chronic abscesses in cattle.

The diagnostic approach employed—clinical observation supported by culture and PCR confirmation—was consistent with routine veterinary practice in the field. While more advanced analyses, such as molecular biotyping and antimicrobial susceptibility testing, could have provided greater epidemiological depth, these were not available within the external diagnostic laboratory at the time of the outbreak. This limitation has been acknowledged, and it underscores the challenges often encountered in clinical case investigations carried out outside of research settings.

One promising approach in controlling such outbreaks involves the use of autovaccines. In this case, healthy animals were vaccinated with an autovaccine derived from the farm-specific *C. pseudotuberculosis* strain. The intervention appeared effective, though further studies are needed to determine long-term protection and efficacy at the herd level. Autovaccines may not provide complete immunity but can be valuable components of integrated disease management strategies [6]. As the vaccine was produced under emergency use authorization in response to the outbreak, its application was primarily part of a herd-level control strategy, not a controlled immunogenicity study. Other efficacy and immunological studies should be carried out in cattle.

The autogenous vaccine, produced from an isolate obtained from affected animals, was used to stimulate herd immunity and prevent further spread. This approach also considered long-term control, as replacement animals were integrated into the vaccination protocol at 12 months of age. The strategy was designed not only to contain the current outbreak but also to serve as a scalable model should the prevalence increase, thus ensuring sustained flock health and productivity. The observed cessation of new clinical cases over a 12-month period post-vaccination supports its field effectiveness, though this does not replace formal efficacy validation.

The decision to employ an autogenous vaccine was made because no commercial vaccines are currently available for use in cattle in Portugal. Vaccination was performed on all animals in the herd, and no local or systemic adverse reactions were reported following administration. Following the intervention, no new clinical cases were observed during the monitoring period. It is important to note, however, that formal immunological or efficacy assessments (e.g., serological monitoring, immune marker quantification, or challenge trials) were not performed, as this was an emergency intervention conducted under field conditions rather than a controlled research trial. Likewise, detailed sterility and safety validation results were not made available by the external laboratory. Therefore, the apparent effectiveness of the autogenous vaccine must be interpreted with caution, as it may also reflect the combined impact of concurrent management measures, including improved biosecurity and hygiene.

Despite these limitations, this experience highlights the potential role of autogenous vaccination as an adjunct tool for outbreak management in atypical hosts. Future studies should aim to evaluate such vaccines under controlled conditions, with comprehensive immunological and safety assessments, to better understand their efficacy and long-term protective capacity.

Effective management of a CL outbreak in cattle requires a multifaceted response: early detection, strict biosecurity, isolation and culling of affected animals, vaccination, and environmental control. In this specific case, immediate implementation of these measures upon outbreak identification was essential to limit the spread and minimize economic losses.

Given the moderate clinical prevalence of approximately 11% in this outbreak, a targeted management approach—comprising selective culling of clinically affected animals, administration of an autogenous vaccine, and reinforcement of biosecurity measures—was implemented to control caseous lymphadenitis (CL) while preserving farm viability. Although more aggressive strategies, such as whole-herd culling, may be considered in severe outbreaks, particularly in sheep where prevalence can reach 40–45% in endemic flocks [21], such measures may be economically unsustainable and could lead to farm closure. The approach adopted in this case reflects established principles of CL control and demonstrates that early detection, herd-specific immunization, and strategic culling can effectively limit disease transmission, even in species where outbreaks are typically more widespread [22]. This strategy provides a scalable and practical model for managing CL in both cattle and small ruminants under varying epidemiological conditions.

Long-term strategies should also be adopted, including regular veterinary monitoring, routine vaccination programs, and fly control measures. Flies are known vectors of *C. pseudotuberculosis* and play a significant role in disease transmission [23], making vector control a critical aspect of prevention.

## 6. Conclusions

This report documents a rare outbreak of caseous lymphadenitis in dairy cattle, expanding the understanding of *Corynebacterium pseudotuberculosis* beyond its typical small-ruminant hosts. While diagnostic and management measures followed standard veterinary practice, their application in this unusual epidemiological context provides relevant field insights. The case underscores the importance of early detection, species-specific autogenous vaccination, and reinforced biosecurity in managing outbreaks while preserving herd productivity. The observed interruption of clinical cases following intervention suggests potential effectiveness, though formal vaccine efficacy studies are still warranted. Future studies are also needed to validate such interventions and to clarify the role of autogenous vaccination in managing emerging or atypical presentations of CL.

This case reinforces the need for proactive, multispecies surveillance and integrated disease control strategies in regions where CL is endemic.

In summary, this case contributes to veterinary literature by documenting a rare outbreak of CL in dairy cattle, detailing its clinical presentation, and describing the management measures applied under field conditions. While the descriptive nature and absence of immunological data limit the analytical depth of the study, the report highlights important lessons for veterinary practitioners and underscores the need for future research on autogenous vaccines against CL, particularly regarding their safety, efficacy, and long-term protective capacity in atypical hosts.

## Data Availability

The data generated during this field study are not publicly available but are available from the corresponding author upon reasonable request.
